# Factors Associated with Composting of Solid Waste at Household Level in Masaka Municipality, Central Uganda

**DOI:** 10.1155/2018/1284234

**Published:** 2018-11-19

**Authors:** Pius Nsimbe, Hilbert Mendoza, Solomon Tsebeni Wafula, Rawlance Ndejjo

**Affiliations:** ^1^Department of Disease Control and Environmental Health, School of Public Health, College of Health Sciences, Makerere University, Kampala, Uganda; ^2^Department of Community Health and Behavioural Sciences, School of Public Health, College of Health Sciences, Makerere University, Kampala, Uganda

## Abstract

The domestic solid waste stream composition of urban settings in many developing countries including Uganda is largely biodegradable in nature, and thus, composting provides the most suitable solid waste management option for these wastes. However, there is limited information about waste composting at the household level and associated determinants in Uganda. A cross-sectional study was employed to collect quantitative data from 368 residents of Masaka municipality, Central Uganda. A semistructured interviewer administered questionnaire was used which assessed knowledge, perceptions, and practices of composting. Data were analysed using STATA 13.0, and binary logistic regression was used to determine the factors that influence composting at the household level. Of the 368 participants, 11.4% were engaged in composting. Factors associated with household level composting were age of 46 years and above (aOR = 2.69, 95% CI = (1.06–6.80)), possession of a garden (aOR = 28.88, 95% CI = (3.85–216.72)), engagement in waste segregation (aOR = 5.56, 95% CI = (2.25–13.86)), and periurban residence (aOR = 3.81, 95% CI = (1.78–8.16)). The practice of composting at the household level was low. This therefore highlights the need for urban authorities to develop initiatives for promoting composting at the household level while considering the identified predictors associated with composting.

## 1. Introduction

Improper management of domestic solid waste remains one of the major environmental health challenges facing most urban centers globally [1]. Urban centers generate at least 1.3 billion tons of solid waste globally per year, and this is projected to increase to 2.2 billion tons by 2025 [2]. These wastes if not well managed can be a source of green gas emissions such as methane from the organic fraction of the waste stream [3]. Poorly disposed or uncollected waste can encourage flooding, air, soil, and water pollution and can have exacerbating impacts on health such as diarrhea, arboviral infections, and respiratory problems [2].

The increasing rates of urbanisation, expansion of urban crop farming, high disposal costs related to landfilling, and incineration have reignited interest in the adoption of composting as a strategy for managing municipal solid wastes in urban areas [4]. Composting provides an environmentally friendly method which not only mitigates problems of atmospheric pollution but also conserves soil fertility and biodiversity [5]. The compost is used by many small-scale farmers in low-income countries as a soil conditioner because it is relatively cheaper compared to commercial mineral fertilisers and is more readily available than animal manure [6]. Composting as a solid waste management approach is very relevant in the highly populated urban areas of low-income countries which are characterized by limited waste handling facilities [7, 8]. However, adoption of composting by households is influenced by several factors such as availability of raw materials [9], training in composting [10], and size of the family [11]. Other socioeconomic factors that influence composting that have been highlighted in other countries include education level, age, and access to information on composting [12].

In Uganda, like in many other low-income countries, over 53% of all the solid waste generated in urban centers is from residential households [13]. Moreover, the solid waste composition in such urban centers is largely organic in nature, and therefore, composting provides the most suitable form of recycling [14]. Composting of these organic wastes is however still small-scale and insignificant, often practiced by a few households and mostly for individual household gardens [15]. In Masaka municipality, small-scale household composting is practiced but not effectively. Effective composting requires special attention to community awareness, financial costs, and infrastructure [8]. Although the use of household solid waste for making compost is a growing form of recycling organic wastes and as an alternative to artificial fertilisers in Uganda, there is paucity of information regarding the adoption of this practice and the factors that might encourage urban households to adopt it. This study aimed at understanding the practice of composting and the associated factors among households in Masaka municipality, Uganda.

## 2. Methods

### 2.1. Study Area and Design

This was a cross-sectional study that utilised quantitative techniques of data collection to collect data on the practice of composting and its associated factors among urban households in Kimaanya-Kyabakuza division, one of the three divisions of Masaka municipality, Masaka district. Masaka municipality has a population of about 103,829 people and a growth rate of 3.6% while Kimaanya-Kyabakuza division has approximately 34,632 persons and 8,862 households [16]. Most people in the division are engaged in business and also practice farming in the less urban areas. The study units were households located in Kimaanya-Kyabakuza division, while residents formed the study participants.

### 2.2. Sample Size and Sampling

A total of 368 residents participated in this study. A simple random sampling technique was used to randomly select 2 villages from each of the two parishes of Kimaanya-Kyabakuza division. The names of all villages in each parish were written on pieces of papers, and the papers were folded and placed in two boxes each for a given parish. The papers in each box were thoroughly mixed by shaking, and two villages from each box were selected one after the other without replacement. Systematic random sampling technique was used to systematically select at least 93 households from each of the four villages. By dividing the population of the villages with the sample size drawn from each village, a sampling interval of households was determined per village. The sampling started off by using the village head's residence as the first household, and the rest were selected using a sampling interval based on the number of households in each village. At the households, the household head was chosen to participate in the study. If the household head was not at home at the time of the study, his/her spouse or the other responsible adult household member was selected to participate in the study. Only one respondent at the household was selected to participate in the study.

### 2.3. Data Collection

Data were collected in January 2017 using an interviewer-administered semistructured questionnaire which was translated into *Luganda*, the local language spoken in the area. Participants were asked questions related to their knowledge, perceptions, and practices on composting. The questionnaire was developed based on reviewed literature on household composting [4, 10, 11]. The data collection tool was pretested in Kitabaazi village, Katwe-Butego division, Masaka municipality, which has many similarities with the study area. Research assistants were trained on appropriate methods of data collection. Data were collected from all households where participants consented to participate in the study.

### 2.4. Data Management and Statistical Analysis

The collected data were entered into EpiData 3.1 (EpiData Association, Denmark) and then exported into STATA 13.0 (Statacorp, Texas, USA) for cleaning and analysis. Frequencies and proportions of variables such as the sociodemographics, knowledge, perceptions, and practices of the participants on composting were run. The outcome variable which was practice of composting was coded as 1 and 0; 1 was for those who practiced composting, and 0 for those who did not. Odds ratios were computed using binary logistic regression to determine factors associated with composting. Simple models consisting of the outcome variable and one predictor were run to obtain crude odds ratios (cORs). Explanatory variables that had a probability (*P*) value ≤0.05 after the simple modeling and those that had biological plausibility were included in the final model using the forward stepwise method to obtain the adjusted odds ratios (aORs).

### 2.5. Ethical Considerations

Ethical approval was obtained from the Makerere University School of Public Health Institutional Review Board. Permission was also sought from Masaka municipality authorities before commencement of the study. Participation in the study was voluntary, and informed consent was obtained from each participant at the time of the study after explaining to them the objectives of the study and how findings would benefit them.

## 3. Results

### 3.1. Sociodemographic Characteristics of Participants

A total of 368 respondents participated in the study representing a 99.5% response rate. About half of the participants were married (199, 54.1%), aged 18–31 years 190 (51.6%), and had a monthly income between 15 and 60 US dollars ($) (190, 51.6%). Majority of the participants were females (253, 68.8%), had post-primary education (222, 60.3%), and were Christians (277, 75.3%) (Table 1).

### 3.2. Awareness, Perceptions, and Practices on Household Composting

Most participants had heard about composting (321, 87.2%), with majority (296, 92.2%) of the participants stating that it was important to engage in household composting. Interestingly, only 42 (11.4%) of the participants were engaged in household composting. More than half (215, 58.4%) had a garden, and 187 (50.8%) were segregating their domestic waste. Two-thirds (243, 66.0%) stated that composting required technical knowledge to engage in, and 247 (67.1%) said it was not worthwhile to compost unless time was sufficient (Table 2).

### 3.3. Factors Associated with Adoption of Household Composting

Participants from periurban locations were 3.8 times more likely to engage in composting as compared to those in the urban locations (aOR = 3.81, 95% CI (1.78–8.16), *Pvalue* = 0.001). Participants aged 46 years and above were 2.7 times more likely to engage in composting (aOR = 2.69, 95% CI (1.06–6.80), *Pvalue* = 0.037). The odds of engaging in composting when the participant had a garden was 28.9 times higher than when they did not (aOR = 28.88, 95% CI (3.85–216.72), *Pvalue* = 0.001). The participants who practiced waste segregation at their homes were 5.6 times more likely to engage in composting (aOR = 5.56, 95% CI (2.25–13.86), *Pvalue* = <0.001) (Table 3).

## 4. Discussion

This study assessed the knowledge, perceptions, practices, and factors associated with composting at the household level. The study findings indicated a low uptake of composting, adequate knowledge, and unsatisfactory perceptions about composting at the household level. Our study also showed that possession of a garden, age of the participant, waste segregation behavior, and periurban residence were significantly associated with engaging in the household level composting.

The study revealed a low proportion of households engaged in composting. Similar studies conducted in urban centers of Kenya, Ethiopia, and the Caribbean islands also documented a minimal engagement of households in composting [17–19]. The low engagement in household composting could be partly attributed to lack of knowledge on the technical aspects of composting like equipment to use and entire composting process as previously highlighted by Hoornweg and colleagues [8]. Another likely explanation for the low engagement in household composting could be due to space constraints since urban residents have small plot sizes. The attitudes towards composting were unsatisfactory, they could partly explain the low engagement in composting although these were not statistically significant. Negative perceptions have been found to be a predictor for composting at the household level [20].

The findings revealed that age was significantly associated with practice of household composting as older respondents were more likely to engage in composting as compared to the younger respondents. This could be probably happen because older participants have more time to invest in composting. Findings from our study corroborate with those from other studies that showed a significant association between composting and old age [20–23], but contradicts those of a study in a Cameroon which indicated that young people were most likely to engage in composting [24]. Engagement of young people in Cameroon was attributed to availability of subsidies and employment opportunities that nongovernmental organisations dealing in waste composting were offering which attracted the highly ambitious and adventurous young population. Our study however suggests the need for promotional programs to capitalize on the opportunity of engaging older people while finding ways to interest younger participants to engage in household composting.

The possession of a garden was a significant explanatory variable associated with household composting. This is consistent with other studies that documented gardening or possession of a garden was a motivating factor for household composting [20, 25]. This association is understandable because people who have gardens may most likely use the compost, the end product of composting in their gardens as a soil conditioner. Using compost in the garden improves soil health by enhancing tilth, increasing water retention, and creating air pockets for meristematic plant root cells to grow [26]. This therefore means that composting as a solid waste management option is more likely to be taken up by households who have gardens than those who lack them.

Households who segregated their waste were more likely to engage in composting. Waste segregation has been known to ease further treatment processes such as composting of wastes [27, 28], and therefore, it is not surprising that those who segregated their waste were more likely to engage in composting. This is consistent with another study in Indonesia which showed that waste segregation was precursor step for successful composting [29]. The process of segregation entails separating the biodegradables from the remaining non-biodegradable solid wastes, so as to ease the process of decomposition of the biodegradable waste. It is therefore vital to scale up solid waste segregation promotional-related programs for effective composting at the household level.

The research findings demonstrated that participants who resided in a periurban area were more likely to engage in composting than their counterparts in the urban areas. This may be attributed to the availability of land in the periurban area which is needed for effective composting and farming. It has been noted that many forms of composting such as trench, pile, and windrow require more space which may be difficult to obtain in the most urban areas yet such space can be available in the periurban areas [30]. Innovative solutions are needed to allow for household composting in land space-constrained urban settings.

A limitation of our study is that the practices on compositing were self-reported, and the research assistants did not directly observe the practice of composting in some households, and in some cases, respondents may have given responses which they thought are acceptable. However this study provides useful insights into the practice of composting and its associated factors. Areas of further research could include conducting studies on composting technologies used and explore factors associated with composting in rural settings.

## 5. Conclusions

Adoption of household composting is still low, and is positively influenced by possession of a garden, practice of segregation of waste, periurban residence, and old age. It is important to provide facilities for waste segregation as a way to encourage composting. Scaling up promotional campaigns on composting and developing strategies for interesting the younger people, land-constrained urban dwellers, and those who do not have gardens would increase engagement in composting at the household level.

## Figures and Tables

**Table 1:** Sociodemographic characteristics of participants.

Sociodemographic characteristics	Number of participants (*n*=368)	Percentage (%)
Residence location		
Urban	280	76.1
Periurban	88	23.9

Age of respondent (years)		
18–31	190	51.6
32–45	115	31.3
>45	63	17.1

Gender		
Male	115	31.2
Female	253	68.8

Marital status		
Single/Never married	101	27.4
Married	199	54.1
Widowed or separated	68	18.5

Level of education		
None or primary	146	39.7
Post-primary	222	60.3

Religion		
Muslims	91	24.7
Christians	277	75.3

Ownership of the dwelling house		
Rent	152	41.3
Complete ownership	216	58.7

Monthly income ($)		
≤15	76	20.6
15–60	190	51.6
>60	102	27.7

**Table 2:** Awareness, perceptions, and practices on household composting.

Variables	Number of participants (*n*=368)	Percentage (%)
*Knowledge on composting*		
Ever heard of composting		
No	47	12.8
Yes	321	87.2
Important to do household composting (*n*=321)		
No	25	7.8
Yes	296	92.2
Knew the type of waste that can be composted		
Did not know (non-biodegradable)	22	6.9
Knew (biodegradable)	299	93.2
Knew the equipment used in composting		
Did not know	168	52.3
Knew (skip, tent, and windrows)	153	47.7

*Perceptions about composting*		
Composting requires a lot of space		
Disagree	111	30.2
Agree	257	69.8
Compost is better than artificial fertiliser		
Disagree	363	98.6
Agree	05	1.4
Composting is not worthwhile		
Disagree	121	32.9
Agree	247	67.1
Composting takes a lot of time		
Disagree	244	66.3
Agree	124	33.7
Composting requires technical knowledge		
Disagree	125	34.0
Agree	243	66.0

*Practice on composting*		
Engaged in composting		
No	326	88.6
Yes	42	11.4
Stored domestic waste at the household		
No	70	19.0
Yes	298	81.0
Had a garden		
No	153	41.6
Yes	215	58.4
Segregated wastes at home		
No	181	49.2
Yes	187	50.8

**Table 3:** Independent predictors for adoption of the household level composting.

Variables	*n* (%)	Crude OR (95% CI)	*P* value	Adjusted OR (95% CI)	*P* value
Residence location					
Urban	280 (76.1)	1		1	
Periurban	88 (23.9)	3.45 (1.77–6.69)^*∗*^	**<0.001**	3.81 (1.78–8.16)^*∗*^	**0.001**

Age of the respondent (years)					
18–31	190 (51.6)	1		1	
32–45	115 (31.3)	2.03 (0.95–4.34)	0.067	1.96 (0.84–4.56)	0.119
>45	63 (17.1)	2.96 (1.29–6.79)^*∗*^	**0.011**	2.69 (1.06–6.80)^*∗*^	**0.037**

Gender					
Male	115 (31.2)	1			
Female	253 (68.8)	0.71 (0.36–1.38)	0.311		

Marital status					
Single/never married	101 (27.4)	1			
Married	199 (54.1)	1.18 (0.55–2.50)	0.674		
Widowed or separated	68 (18.5)	0.79 (0.28–2.25)	0.662		

Level of education					
None or primary	146 (39.7)	1			
Postprimary	222 (60.3)	1.08 (0.56–2.09)	0.824		

Religion					
Muslims	91 (24.7)	1			
Christians	277 (75.3)	2.12 (0.86–5.20)	0.102		

Ownership of the dwelling house					
Rent	152 (41.3)	1			
Complete ownership	216 (58.7)	1.16 (0.60–2.25)	0.654		

Monthly income ($)					
≤15	76 (20.6)	1			
15–60	190 (51.6)	1.53 (0.59–3.93)	0.379		
>60	102 (27.7)	1.86 (0.68–5.08)	0.228		

Knowledge factors					
Ever heard of composting					
No	47 (12.8)	1			
Yes	321 (87.2)	2.03 (0.60–6.85)	0.255		

Important to do household composting (*n*=321)					
No	25 (7.8)	1			
Yes	296 (92.2)	0.40 (0.15–1.07)	0.067		

Knew the type of waste that can be composted					
Did not know (non-biodegradable)	22 (6.9)	1			
Knew (biodegradable)	299 (93.2)	1.42 (0.32–6.31)	0.647		

Knew the equipment used in composting					
Do not know	168 (52.3)	1			
Knew (skip, tent, and windrows)	153 (47.7)	1.33 (0.68–2.60)	0.411		

Perceptions factors					
Composting requires a lot of space					
Disagree	111 (30.2)	1			
Agree	257 (69.8)	1.96 (0.88–4.39)	0.100		

Compost is better than artificial fertiliser					
Disagree	363 (98.6)	1			
Agree	05 (1.4)	1.96 (0.21–18.0)	0.551		

Composting is not worthwhile					
Disagree	121 (32.9)	1			
Agree	247 (67.1)	0.98 (0.49–1.93)	0.947		

Composting takes a lot of time					
Disagree	244 (66.3)	1			
Agree	124 (33.7)	0.87 (0.43–1.74)	0.690		

Composting requires technical knowledge					
Disagree	125 (34.0)	1			
Agree	243 (66.0)	1.17 (0.58–2.33)	0.661		
Had a garden					
No	153 (41.6)	1		1	
Yes	215 (58.4)	35.8 (4.87–263.49)^*∗*^	**<0.001**	28.88 (3.85–216.72)^*∗*^	**0.001**
Segregated wastes at home					
No	181 (49.2)	1		1	
Yes	187 (50.8)	5.72 (2.47–13.26)^*∗*^	**<0.001**	5.58 (2.25–13.86)^*∗*^	**<0.001**

^*∗*^Statistically significant *P* value <0.05.

## Data Availability

The dataset used to support the findings of this study is available from the corresponding author upon request.
